# Aerogels of 1D Coordination Polymers: From a Non-Porous Metal-Organic Crystal Structure to a Highly Porous Material

**DOI:** 10.3390/polym8010016

**Published:** 2016-01-15

**Authors:** Adrián Angulo-Ibáñez, Garikoitz Beobide, Oscar Castillo, Antonio Luque, Sonia Pérez-Yáñez, Daniel Vallejo-Sánchez

**Affiliations:** Departamento de Química Inorgánica, Facultad de Ciencia y Tecnología, Universidad del País Vasco, UPV/EHU, Apartado 644, E-48080 Bilbao, Spain; aangulo017@ikasle.ehu.eus (A.A.-I.); antonio.luque@ehu.eus (A.L.); sonia.perez@ehu.eus (S.P.-Y.); daniel.vallejo@ehu.eus (D.V.-S.)

**Keywords:** aerogel, porosity, metal-organic aerogel, coordination polymer, metal-organic framework, MOA, PCP, MOF

## Abstract

The processing of an originally non-porous 1D coordination polymer as monolithic gel, xerogel and aerogel is reported as an alternative method to obtain novel metal-organic porous materials, conceptually different to conventional crystalline porous coordination polymer (PCPs) or metal-organic frameworks (MOFs). Although the work herein reported is focused upon a particular kind of coordination polymer ([M(μ-ox)(4-apy)_2_]_n_, M: Co(II), Ni(II)), the results are of interest in the field of porous materials and of MOFs, as the employed synthetic approach implies that any coordination polymer could be processable as a mesoporous material. The polymerization conditions were fixed to obtain stiff gels at the synthesis stage. Gels were dried at ambient pressure and at supercritical conditions to render well shaped monolithic xerogels and aerogels, respectively. The monolithic shape of the synthesis product is another remarkable result, as it does not require a post-processing or the use of additives or binders. The aerogels of the 1D coordination polymers are featured by exhibiting high pore volumes and diameters ranging in the mesoporous/macroporous regions which endow to these materials the ability to deal with large-sized molecules. The aerogel monoliths present markedly low densities (0.082–0.311 g·cm^−3^), an aspect of interest for applications that persecute light materials.

## 1. Introduction

The unique properties, the structural beauty and the multiple functionalities that porous coordination polymers (PCPs or MOFs) match up, have prompted a myriad of research works which up to date have been the focus of the scientific community devoted to chemistry, physics, materials science or to any other bordering research area [1,2]. Despite the variety of approaches and topics dealt with in these works, all of them share the same cornerstone: porosity. The topology of the coordination network and of the underlying porosity are governed by the principles of modular chemistry where the design of porous crystalline materials is rationalized on the basis of secondary building units (SBUs) and politopic organic linkers [3,4]. Regarding the pore size of MOFs, most of the reported crystal structures enclose a microporous network, while the examples of mesoporous MOFs lie on the lower boundary of mesoporosity (2–10 nm) [5,6,7,8]. The factor limiting the pore size is the extension of the organic linker, the increasing size of which implies not only a synthetic challenge but also can weaken the stability of the crystal structure or lead to interpenetrated structures. The small pore sizes of MOFs are specially appealing in storage and separation of weakly interacting small molecules, where the narrowness of the cavities reinforce the adsorbate-adsorbent interactions. Nevertheless, reduced pore sizes imply in several cases disadvantages such as prolonged diffusing times and limited accessibility, particularly in applications dealing with large molecules such as, for example, separation, catalysis or sensing of biomolecules or of other non-polymeric macromolecules [9].

In this sense, to overcome the crystal structure limited pore size, exo- and endo-templates have been employed in MOFs synthesis rendering materials that bring together the intrinsic micro-/mesoporosity of the coordination network and the extrinsic meso-/macroporosity arising from the removal of the template [10,11,12,13]. For instance, a handful of published studies illustrate how the MOF synthesis in microemulsion of surfactants and swelling agents allows for the modulation of the extrinsic pore size and the total porosity of the material [14,15,16,17,18,19,20].

Another possibility to incorporate extrinsic porosity to an MOF lies in the supercritical drying of a colloidal gel (MOG: Metal-Organic Gel) which leads to aerogels (MOA: Metal-Organic Aerogel) featured by containing high porosity values and extremely low density. Despite studies on aerogels of ceramic and organic materials having a dilated trail that have even lead them to a successful commercialization [21], examples of MOFs aerogels are relatively recent and scarce [22,23,24,25]. As a consequence, the synthesis of MOF aerogels is an area that still implies significant future development. One of the challenges to afford new MOAs is to precisely understand the factors governing the gelation process in order to achieve robust enough gels with low syneresis, as the experimental conditions of one metal-organic system are not applicable in another one [26]. In any case, the anisotropic growth of the colloid can be considered as a general rule that favours the gel formation.

**Figure 1:**
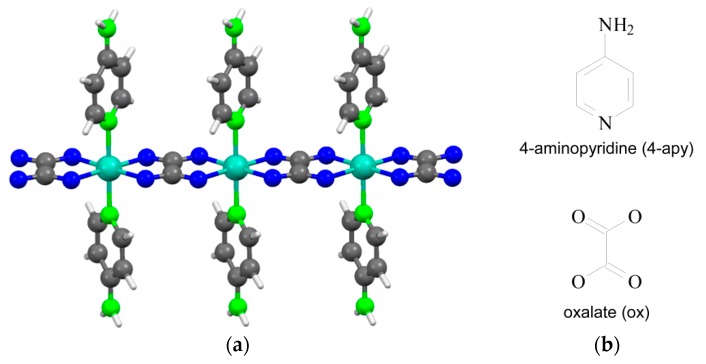
(**a**) Perspective view of the polymeric chain in the compound [Ni(μ-ox)(4-apy)_2_]_n_ (color code: C (gray); H (white); N (green) Ni (turquoise), O (blue). CSD code: UCOZOB); (**b**) Chemical diagrams of the ligands.

Bearing in mind the latter issue and all the above statements regarding MOFs, in order to prepare aerogels of coordination polymers, herein we have selected a metal-organic 1D system with a dual purpose: (1) the one-dimensional feature of the coordination complex can prompt an anisotropic growth of colloidal crystals favouring their physical aggregation (crosslinking through non-covalent interactions) and gel formation, as it occurs in the Cu(II)-oxalate metallogels reported by Saha *et al.* [27]; (2) preparing aerogels of originally non-porous coordination polymers enhances the possibilities of metal-organic materials, as the porosity is achieved without the requirements imposed by the crystal engineering of conventional MOFs. The cited 1D polymer presents the formula [M(μ-ox)(4-apy)_2_]_n_ (M: Co(II), Ni(II), Cu(II); ox: oxalate; 4-apy: 4-aminopyridine). Its synthesis and crystal structure (Figure 1) was reported in a previous study by our group [28], and thus, its selection is also due to the accumulated synthetic experience on metal/oxalate/*N*-ligand systems which is decisive in optimization of the gelation process.

## 2. Experimental Section

All reagents and solvents were commercially available and used directly without any further purification.

### 2.1. Preparation of Metal-Organic Gels, Xerogels and Aerogels

**Scheme 1:**
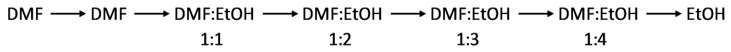
Steps followed during the solvent exchange to remove the unreacted species from the gel and to replace the synthesis solvent by ethanol.

For obtaining metal-organic gels (also called, metallogels), 1 mmol of M(NO_3_)_2_·6H_2_O (M: Co(II) or Ni(II)) is dissolved in 2 mL of *N*,*N*-dimethylformamide (DMF). A second solution consisting of 1 mmol of oxalic acid, 2 mmol of 4-aminopyridine, 2 mmol of triethylamine and 2 mL of DMF was prepared. To ensure an immediate mixture of all the reagents, the metal ion solution is added at once upon the solution containing the ligands and the base which is subjected while mixing to a continuous sonication using an ultrasound bath (ULTRASONS-H, Selecta, Barcelona, Spain). A few minutes later, the resulting mixture showed a gel appearance whose colour (pink and turquoise for Co(II) and Ni(II), respectively) matches the expected for the previously reported compounds. Despite the 1D coordination polymer can be also prepared with Cu(II), all the gellation trials with this transition metal were unsuccessful rendering fine precipitates instead a gel. As prepared samples were aged during 24 h, and thereafter, they were thoroughly washed firstly by immersing in pure DMF to remove the unreacted species and secondly by successive solvent exchanges using DMF/ethanol mixtures and pure ethanol to replace the solvent. The successive solvent exchanges are depicted in Scheme 1.

In each step the contact between solvent and gel was prolonged for 24 h in order to ensure an efficient exchange. Xerogels (xeroCo and xeroNi for Co and Ni compounds, respectively) were prepared subjecting the metallogels to open atmosphere drying. To prepare the aerogels (aeroCo and aeroNi), a E3100 critical point dryer from Quorum Technologies (Lewes, United Kingdom) equipped with gas inlet, vent, and purge valves and with a thermal bath was employed. Firstly, the metallogel is immersed in liquid CO_2_ at 20 °C and 50 atm for 1 h after the exchanged ethanol is removed through the purge valve. This process is repeated five times. Subsequently, the sample is dried under supercritical conditions increasing the temperature and pressure to 38 °C and 85–95 bar. Finally, under constant temperature (38 °C) the chamber is slowly vented up to atmospheric pressure.

### 2.2. Physical Measurements

Elemental analyses (C, H, N) were performed on an Euro EA elemental analyzer (Eurovector, Milan, Italy), whereas the metal content was determined by inductively coupled plasma atomic emission spectrometer (ICP-AES) from Horiba Yobin Yvon Activa (Kyoto, Japan). The IR spectra (KBr pellets) were recorded on a FTIR 8400S Shimadzu spectrometer (Shimadzu, Kyoto, Japan) in the 4000–400 cm^−1^ spectral region. Thermal analyses (TG/DTA) were performed on a TA Instruments SDT 2960 thermal analyser (TA Instruments, New Castle, DE, USA) from room temperature to 800 °C in a synthetic air atmosphere (79% N_2_/21% O_2_) with a heating rate of 5 °C/min. Powder X-ray diffraction (PXRD) patterns were collected on a Phillips X'PERT powder diffractometer (Panalytical, Eindhoven, The Netherlands) with Cu-Kα radiation (λ = 1.54060 Å) over the range 5° < 2θ < 70° with a step size of 0.02° and an acquisition time of 2 s per step at 25 °C. For comparative purposes simulated PXRD patterns were calculated from the original crystal structure using the Mercury 3.0 (CCDC, Cambridge, United Kingdom), crystal Structure visualisation, exploration and analysis tool [29]. The apparent density values of the dry monoliths were geometrically determined using an analytical balance and a digital caliper (PCE Ibérica S.L., Tobarra, Spain). Dinitrogen (77 K) and carbon dioxide (273 and 298 K) physisorption data were recorded on activated samples (vacuum at 100 °C for 4 h) with a Quantachrome Autosorb-iQ-MP (Quantachrome Instruments, Boynton Beach FL, USA). The specific surface area was calculated from the adsorption branch in the relative pressure interval of 0.01–0.10 using the Brunauer–Emmett–Teller (BET) method.

## 3. Results and Discussion

### 3.1. Gelation and Appearance of Gels, Xerogel and Aerogel

**Figure 2:**
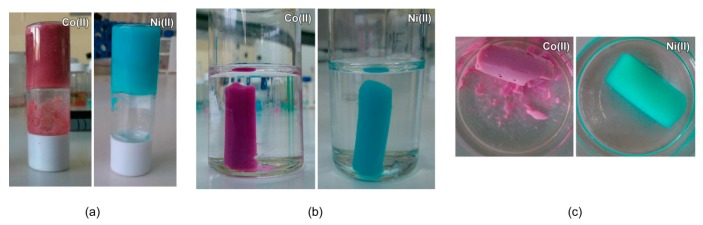
(**a**) As prepared gel; (**b**) gel washing; and (**c**) aerogel.

The gelation conditions were optimized by using the test-tube inversion method: a material is considered a gel if it does not suffer deformation after inverting the vial where it is contained. For this purpose, metal ion source (nitrate, chloride or acetate), reagent concentration and type of solvent were varied in order to understand the most influential factors. The reagent molar ratio was set to 1:1:2 (metal:ox:4-apy) in order to fit the stoichiometric relation in the compound. In terms of gel stiffness, the best results were obtained for nitrate salts in DMF at concentration within 0.2–0.3 M. Greater concentrations led to particulate precipitates, while lower ones provided too feeble gels to pass the test-tube inversion test. Results obtained in water and alcoholic solvents were also not satisfactory, rendering in all cases a particulate precipitate. The addition of the base triethylamine to originate a basic media was also crucial, in such a way that its avoidance or replacement by other base (NaOH) resulted in feeble gels and particulate precipitate, respectively. Figure 2a shows the gel prepared under selected conditions (see Experimental Section). The stiffness of the gels allowed us to wash them (Figure 2b) by a solvent exchange process in which unreacted reagents were removed and the DMF was replaced by ethanol. Supercritical drying of both gels produced well shaped monolithic aerogels (Figure 2c). The Co(II) specimen (aeroCo) was pink colored and exhibited chalk appearance. Moreover, it behaved as a feeble and brittle material when manipulated. Conversely, the turquoise colored Ni(II) specimen (aeroNi) was somewhat translucent, which matches better with the appearance of common silica aerogels and it allows us to envisage a material of lower density and built from smaller particles.

Shrinkage degree of the gels during aerogel and xerogel formation and density values of the dry monoliths were measured (Table 1 and Tables S1 and S2 (Supplementary Material)), as they provide a clue to the success of the aerogel formation and the presumable porosity. In the case of aerogels, Ni(II) sample presents a negligible contraction which implies that all volume corresponding to the solvent has been retained as pore volume in the dry material. Co(II) sample reduces its initial volume by 57% during the supercritical drying, suggesting a marked reduction of the final porosity. Equally, the shrinkage degree explains also the density differences; the lightness of Ni(II) specimen is notable. Both xerogels showed a severe contraction (>90%) during their formation, leading to materials of higher densities.

**Table 1:** Shrinkage degrees and density values for aerogels and xerogels monoliths.

Sample	Shrinkage (%)	Density (g·cm^−3^)
aeroCo	57.0	0.311
xeroCo	92.5	1.783
aeroNi	<0.1	0.082
xeroNi	93.6	1.281

### 3.2. Chemical Analysis and Molecular Structure

**Figure 3:**
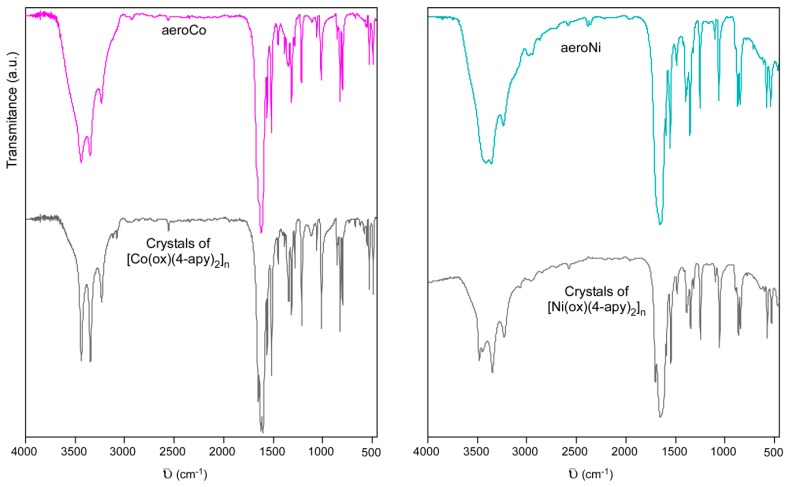
FTIR spectra for Co(II) and Ni(II) aerogels and crystals of [M(ox)(4-apy)_2_]_n_ (M(II): Co, Ni).

Fast growing conditions required for the gelation of these materials leads to faulty, almost amorphous, crystal structures in which some of the 4-aminopyridine ligands sited at the apical positions of one-dimensional metal(II)-oxalate chains, can be unoccupied or replaced by water and DMF molecules. When compared to the 1D-[M(ox)(4-apy)_2_]_n_ pristine structure, the elemental analyses and themogravimetric measurements (see Tables S3–S6 and Figures S1 and S2, Supplemenatry Material) allowed us to define the 4-aminopyridine deficient formulas Co(ox)(4-apy)_1.4_(DMF)_0.2_(H_2_O)_0.4_ and Ni(ox)(4-apy)_1.6_(DMF)_0.3_(H_2_O)_0.7_, in which it is difficult to precisely determine the state of DMF and water molecules (coordinated, embedded in between the polymeric chain or embedded in the intercrystallite spaces). In any case, the overall molecular structure is supported by good agreement among the IR spectra of the Ni(II) and Co(II) aerogel and xerogels and that correspond to single-crystals of the pristine compounds (Figure 3), where characteristic vibration modes of the bis-bidentate oxalate ligand and 4-aminopyridine are identified (see Tables S7 and S8 and Figure S3, Supplemenatry Material). It is noteworthy that the water content in aerogel and xerogel samples seems to broaden the bundle of peaks sited between 3500–3000 cm^−1^ and corresponding to 4-aminopyridine N–H and C–H stretching modes.

**Figure 4:**
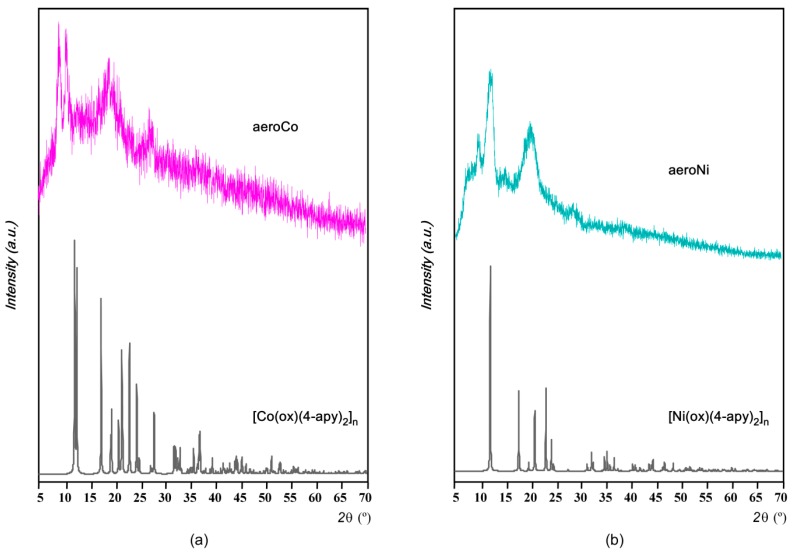
Powder X-ray diffraction (PXRD) experimental patterns on aerogels and the ones simulated from the crystal structures of 1D-[M(ox)(4-apy)_2_]_n_ for (**a**) Co(II) and (**b**) Ni(II) (CSD codes: UCOZIV and UCOZOB, respectively).

On the other hand, PXRD measurements on aerogel and xerogel samples revealed diffraction patterns corresponding to scarcely crystalline samples as can be expected from the above discussion (Figure 4). In spite of that, the observed weak peaks do not properly fit the diffraction patterns simulated from the crystallographic data files of 1D-[M(ox)(4-apy)_2_]_n_ (M: Ni(II), Co(II)) compounds, which suggest discrepancies in the crystal structures probably prompted by the 4-aminopyridine deficiency. In fact, subtle changes in the metal-ligand bond distances and angles also prompt chain to chain relative displacements and as a result, different crystal systems are obtained for the pristine compounds ([Co(ox)(4-apy)_2_]_n_: monoclinic, *C*2/*c*; [Ni(ox)(4-apy)_2_]_n_: orthorhombic, *Fddd*).

Finally, it must be pointed out that compared to xerogels, the autoignition of aerogels takes place at noticeably lower temperatures (aeroCo and aeroNi: 285 and 360 °C; xeroCo and xeroNi: 326 and 390 °C). Such differences between the stabilities of aerogels and xerogels is explained by the greater porosity and surface area presumed for the first one, as it would facilitate the evacuation of volatile products and accelerate the decomposition kinetics. In fact, the autoignition value of the xerogels that present negligible surface areas are close to those reported for crystals of [M(ox)(4-apy)_2_]_n_ (330 and 390 °C for Co(II) and Ni(II) compounds, respectively).

### 3.3. Microstructural Characterization

**Figure 5:**
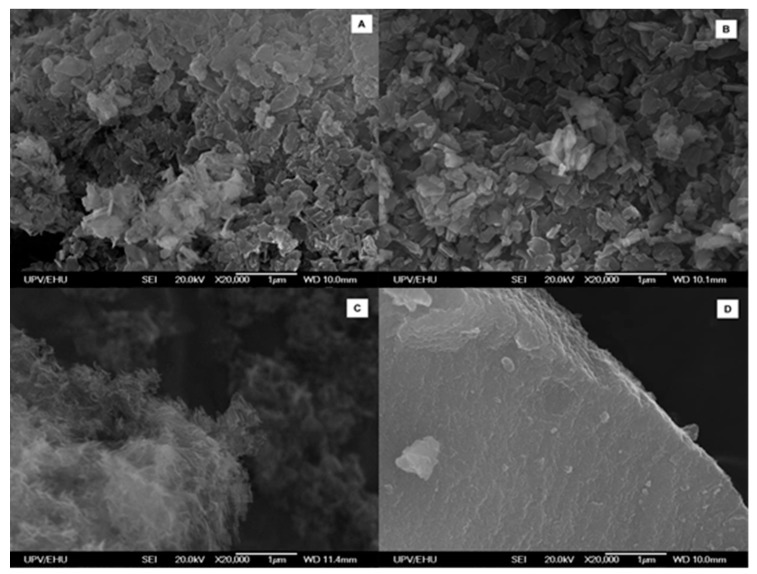
SEM images at 20 kX of aeroCo (**A**); xeroCo (**B**); aeroNi (**C**) and xeroNi (**D**).

The microstructural study is performed on the basis of scanning electron microscopy (SEM) and gas adsorption isotherm analyses. Figure 5 gathers SEM micrographs for aerogel and xerogel samples. The samples of aeroCo and aeroNi are comprised of an assembly of submicrometric lamellar crystals that enclose a significant porosity. In contrast, the previously reported Cu/ox MOGs consisted of crosslinked 20–45 nm fibers of high aspect-ratio [27], and thus the lamellar shape of the particles comprising M/ox/4-apy MOGs can be attributed to interchain hydrogen bonding established between the apical and bridging ligands, as it can appreciate in the original crystal structures [28]. It must be emphasized that, according to these images, aeroNi is comprised of thinner crystals and it exhibits more porous microstructure which matches the shrinkage of the metallogels during aerogel formation (aeroCo: 57%; aeroNi: <0.1%) and with the density values of the monoliths (aeroCo: 0.311 g·cm^−3^; aeroNi: 0.082 g·cm^−3^). Apart from that, the smaller size of the crystals observed for Ni(II) also explains its translucent appearance when compared to the rough and opaque surface of Co(II) aerogel perceived at visual inspection. Due to the collapse taking place during the open atmosphere drying, both xerogels present a more compact microstructure than their aerogel analogues.

**Figure 6:**
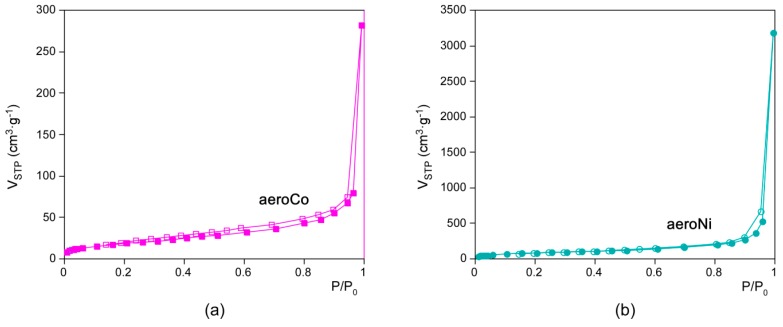
N_2_ adsorption isotherms for (**a**) aeroCo and (**b**) aeroNi samples (filled symbol: adsorption branch; open symbol: desorption branch).

**Figure 7:**
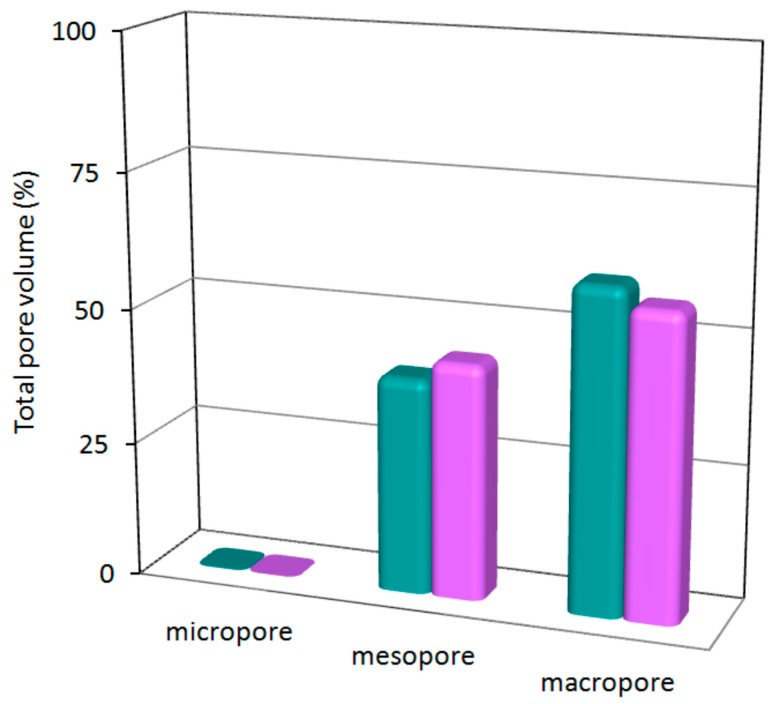
Porosity distribution within the different pore regions for aeroNi (turquoise bars) and aeroCo (pink bars).

The porosity of the aerogels was further analyzed by adsorption isotherms of N_2_ measured at 77 K (Figure 6). Subtracted porosity data is gathered in Table 2. In concordance with the stated above, surface area and pore volume values are significantly greater for aeroNi due to its minor shrinkage. Although origin of the porosity of MOFs and MOAs is not the same, the pore volume value of aeroNi (4.92 cm^3^·g^−1^) is comparable to the top values of MOFs [30]. In contrast, the surface area (311 m^2^/g) is far away from most outstanding MOFs, but it is comparable to many MOFs and zeolites with moderate surface area values. On the other hand, the pore volume and pore size (mode: 40 nm) can be considered as relatively high; in fact, the pore size far exceeds the maximum value reported for an MOF up to date (<10 nm). The latter feature provides to the aerogels of coordination polymer the ability to deal with molecules of greater size, such as proteins or other complex biological assemblies (enzymes, antibodies, polypeptides, *etc.*). It must be considered that, contrary to the MOFs, the porosity coming from the aerogel microstructure is featured by a wide pore size distribution, and according to BJH fitting of the adsorption data (see Figure S4, Supplemenatry Material). Despite the mode value in both aerogels being sited at the mesoporous region, the contribution of macropores to the total pore volume is somewhat greater (Figure 7), which can also be inferred from the predominant type II shape of the adsorption isotherms.

**Table 2:** Porosity data for aeroCo and aeroNi *.

Sample	aeroCo	aeroNi
*S*_BET_ (m^2^·g^−1^)	68.3	311.2
*S*_micro_ (m^2^·g^−1^)	0.0	33.0
*S*_meso/macro_ (m^2^·g^−1^)	68.3	278.2
*V*_T_ (cm^3^·g^−1^)	0.44	4.93
*V*_micro_ (cm^3^·g^−1^)	0.00	0.01
*V*_meso/macro_ (cm^3^·g^−1^)	0.43	4.92
*D*_pore_ (nm)	37	32
Porosity (%)	14	40

* S_BET_ stands for BET specific surface area. Micropore surface area (*S*_micro_) and volume (*V*_micro_) are estimated from the *t*-plot calculation. Meso-/macroporous area and volume (*S*_meso/macro_, *V*_meso/macro_) are calculated by substracting the microporous contribution total area and volume.

**Figure 8:**
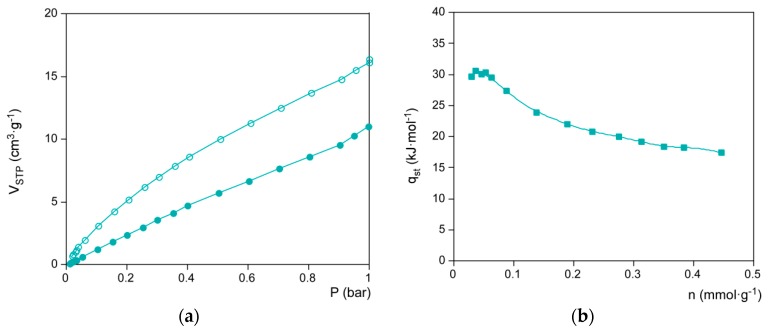
(**a**) CO_2_ adsorption isotherms for aeroNi sample at 273 (open-circles) and 298 K (filled-circles); (**b**) Isosteric heat values *versus* CO_2_ uptake.

With the aim of completing the characterization of the porosity, CO_2_ adsorption isotherm were collected at 273 and 298 K on most porous aeroNi sample in order to estimate the isosteric heats of adsorption (*Q*_st_) that renders this kind of materials (Figure 8). The *Q*_st_ value has been calculated using the Clausius–Clapeyron equation [31,32], which, in the limit of zero loading, approaches 31.9 kJ·mol^−1^. This value is lower than those reported for the most strongly interacting MOFs such as Cr-MIL-100 (62 kJ·mol^−1^) [33], Cr-MIL-101 (44 kJ·mol^−1^) [33], and Mg-MOF-74 (42 kJ·mol^−1^) [34], but of the same order of well-known Al-MIL-53 (35 kJ·mol^−1^) [35], Ni-STA-12 (35 kJ·mol^−1^) [36], Zn2(dobdc) (26 kJ·mol^−1^) [37] and Cu-HKUST-1 (29 kJ·mol^−1^) [38]. In any case, it can be regarded as a relatively high value for a mesoporous material [39] probably due to the presence of the exocyclic amino groups decorating the surface of the nanocrystals, which has been demonstrated to effectively interact with CO_2_ [40,41].

## 4. Conclusions

The current work has reported the processing of an originally non-porous coordination polymer as monolithic gel, xerogel and aerogel. Although the work herein reported is focused on a particular kind of coordination polymer ([M(μ-ox)(4-apy)_2_]_n_, M: Co(II), Ni(II)), the results are of interest in the field of porous materials and of MOFs. One of the most remarkable results arises from the possibility of dispensing of the prerequisites imposed by the reticular design of MOFs, which, as a result, implies that this approach to prepare porous coordination polymers is in principle extensible to any other metal-organic system. It must be emphasized that meeting porosity and functionality in MOFs is not always a straightforward issue. In this sense, one must consider that there are many polymers with appealing electrical, magnetic and optic properties whose crystal structures lack any porosity arising from the connectivity of coordination network [42]. As a consequence, there is still an exciting chance to process such kinds of coordination polymer as aerogels in order to access a new generation of stimuli-response materials. Another singular aspect is related with the pore size of the coordination polymer aerogels which far exceed that of MOFs, and it endows them the ability to deal with greater molecules, opening also the opportunity towards new applications of this kind of materials, for instance, separation, transportation or processing of macromolecules. The achievable low densities (aeroNi: 0.082 g·cm^−3^) is another aspect of interest in materials science, especially when a certain application requires light materials. Finally, it is noteworthy that the applications of MOFs demand that users process them as monolithic materials, which is still a mayor challenge because the cohesion of the monolith requires binders that detriment the porosity. Therefore, preparing coordination polymers as monolithic gels and aerogels brings out another outstanding point of the synthetic approach herein reported, as it lacks of any additive or binder.

## Supplementary Materials

The following are available online at www.mdpi.com/2073-4360/8/1/16/s1.

## Abbreviations

PCP: Porous Coordination Polymer

MOF: Metal-Organic Framework

MOG: Metal-Organic Gel

MOA: Metal-Organic Aerogel
