# Supplementary material for: Aerogels of 1D Coordination Polymers: From a Non-Porous Metal-Organic Crystal Structure to a Highly Porous Material
Source: Polymers (Basel). 2016 Jan 15;8(1):16. doi: 10.3390/polym8010016 (PMC6432558; doi:10.3390/polym8010016)
Supplement: Supplementary file 1 [file polymers-08-00016-s001.pdf]

Supplementary Materials: Aerogels of 1D Coordination Polymers: From a Non-Porous Metal-Organic Crystal Structure to a Highly Porous Material

Adrián Angulo-Ibáñez, Garikoitz Beobide, Oscar Castillo, Antonio Luque, Sonia Pérez-Yáñez and Daniel Vallejo-Sánchez

1. Dimensions of Gel, Xerogel and Aerogel Specimens

Dimension changes taking place during open atmosphere drying of gel cylinders (xerogels) and during supercritical drying (aerogels) are gathered in Tables S1 and S2.

Table S1. Gel and xerogel dimensions of Co(II) and Ni(II) samples.

	gelNi	xeroNi	Shrinkage (%)	gelCo	xeroCo	Shrinkage (%)
Length (cm)	2.0	0.8	60.0	1.5	0.7	53.3
Diameter (cm)	1.5	0.6	60.0	1.5	0.6	60.0
Volume (cm^3)	3.53	0.226	93.6	2.65	0.198	92.5

Table S2. Gel and aerogel dimensions of Co(II) and Ni(II) samples.

	gelNi	aeroNi	Shrinkage (%)	gelCo	aeroCo	Shrinkage (%)
Length (cm)	1.6	1.6	0.0	1.3	1.1	15.4
Diameter (cm)	0.6	0.6	0.0	0.7	0.5	28.6
Volume (cm^3)	0.45	0.45	0.0	0.50	0.22	57.0

2. Chemical and Thermogravimetric Analysis

Elemental content found in the chemical analyses are shown in Tables S3 and S4 together with the expected values for the 4-aminopyridine deficient formulas $Co(ox)(4\text{-}apy)_{1.4}(DMF)_{0.2}(H2O)_{0.4}$ ($Co(C_2O_4)(C_5H_6N_2)_{1.4}(C_3H_7NO)_{0.2}(H_2O)_{0.4}$) and $Ni(ox)(4\text{-}apy)_{1.6}(DMF)_{0.3}(H_2O)_{0.7}$ ($Ni(C_2O_4)(C_5H_6N_2)_{1.6}(C_3H_7NO)_{0.3}(H_2O)_{0.7}$). Molecular formulas of aeroCo and aeroNi fit mass losses observed in the thermogravimetric analyses. Results of the thermogravimetric analyses are presented in Tables S5 and S6, plots of the TG and DTA curves are shown in the in Figure S.1. Residues were identified by PXRD measurements.

It must be pointed out that despite aerogel and xerogel are essentially equal in terms of chemical composition and molecular assembly, they discord in the way they decompose due to the microestructural differences. DMF molecules bind stronger to Ni(II) than to Co(II), and thus completing their evacuation requires of higher temperatures in aeroNi sample, lengthening the stage corresponding to solvent removal up to 230 °C.

Table S3. Results on the elemental analysis for aeroCo.

$Co(C_2O_4)(C_5H_6N_2)_{1.4}(C_3H_7NO)_{0.2}(H_2O)_{0.4}$	C	H	N	O	Co
% Experimental	38.33	3.81	13.62	23.94	20.30
% Theoretical	38.36	3.55	13.98	24.49	19.61

Table S4. Results on the elemental analysis for aeroNi.

$Ni(C_2O_4)(C_5H_6N_2)_{1.6}(C_3H_7NO)_{0.3}(H_2O)_{0.7}$	C	H	N	O	Ni
% Experimental	39.21	4.34	14.34	24.71	17.40
% Theoretical	39.45	3.98	14.77	24.11	17.67

Table S5. Thermogravimetric analysis for aeroCo.

Stage	T_i	T_f	T_{peak}	Δm(%)	ΔH	$\Sigma\Delta m$(%)	$\Sigma\Delta m(\%)_{theor}$
1	20	150	36	7.7	Endo	7.7	7.3 [$-(H_2O)_{0.4}(DMF)_{0.2}$]
2	160	315	285/300	65.0	Exo	72.7	73.3 [Residue: 1/3Co_3O_4]

T_i: onset temperature; T_f = endset temperature; T_{peak} = DTG peak temperature; Δm(%) = weight loss percentage at each step; ΔH = enthalpy of each stage; $\Sigma\Delta m$(%) = cumulative weight loss percentage; $\Sigma\Delta m(\%)_{teor}$ = theoretical cumulative weight loss percentage calculated from the formula of aeroCo.

Table S6. Thermogravimetric analysis for aeroNi.

Stage	T_i	T_f	T_{peak}	Δm(%)	ΔH	$\Sigma\Delta m$(%)	$\Sigma\Delta m(\%)_{theor}$
1	20	230	40	11.0	Endo	11.0	10.4 [$-(H_2O)_{0.7}(DMF)_{0.3}$]
2	230	370	360	66.3	Endo/Exo	77.3	78 [Residue: NiO]

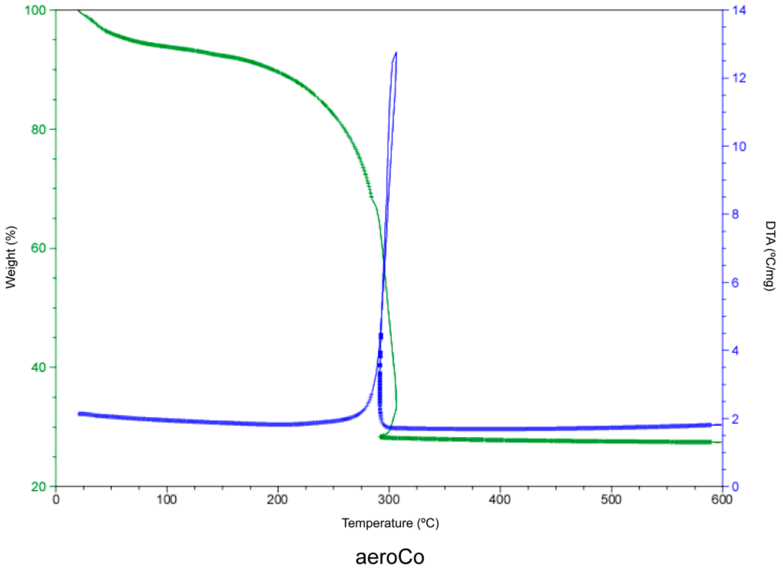

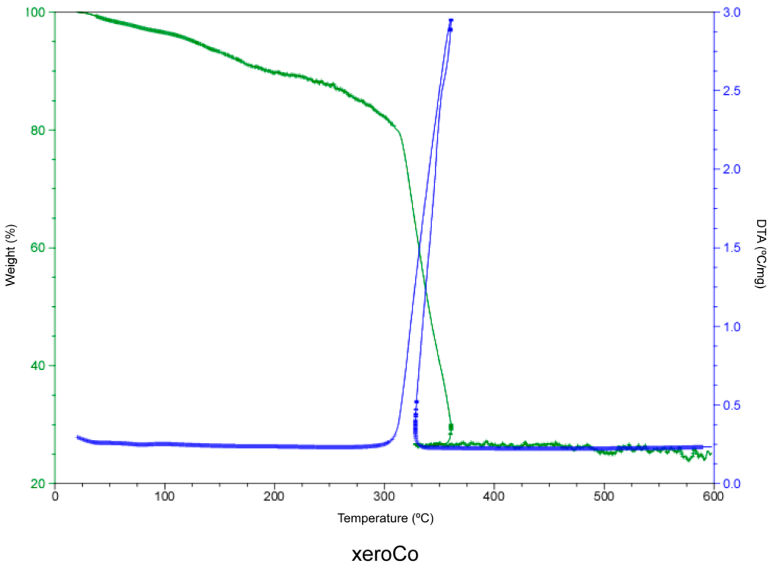

Figure S1. Thermogravimetric (TG: green line) and differential thermal analysis (DTA: blue line) curves for aerogel and xerogel samples.

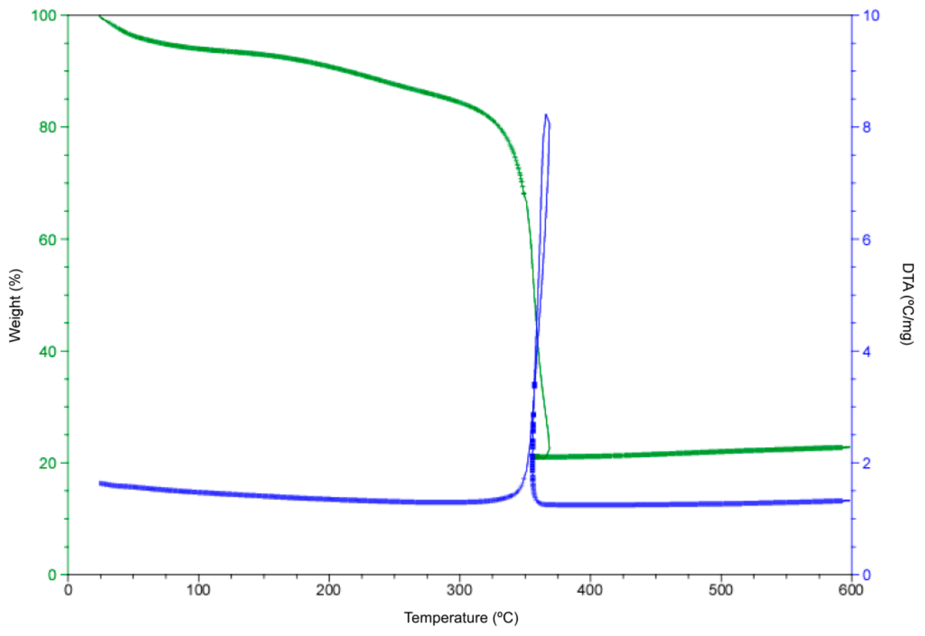

aeroNi

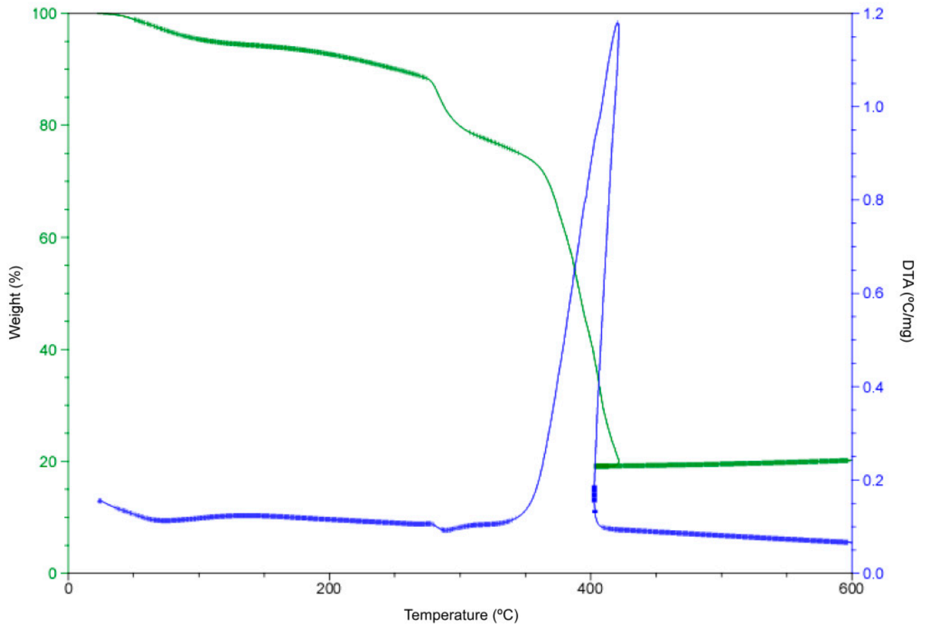

xeroNi

Figure S2. Thermogravimetric (TG: green line) and differential thermal analysis (DTA: blue line) curves for aerogel and xerogel samples.

3. Ftir Spectra

FTIR spectra of aerogel and xerogel sample are shown in Figure S2 while the corresponding vibration modes are gathered in Tables S7 and S8.

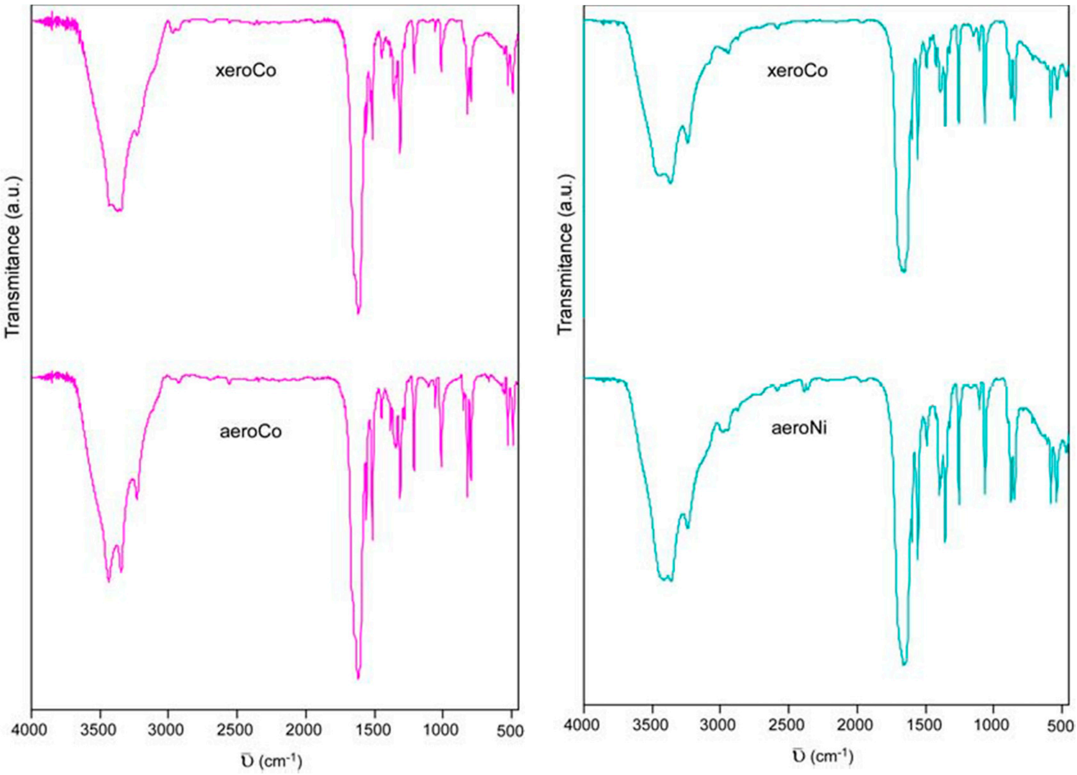

(**a**) (**b**)

Figure S3. FTIR spectra for (**a**) Co(II) and (**b**) Ni(II) aerogels and xerogels.

Table S7. FTIR peaks (cm^{-1}) and corresponding vibration modes for aeroCo, xeroCo and single crystals of $[Co(ox)(4\text{-}apy)_2]_n$ [a].

aeroCo	xeroCo	$[Co(ox)(4\text{-}apy)_2]_n$	Vibration Mode
3,440 s	3,437 s	3,437 s	
3,341 s	3,349 s	3,349 s	υ N–H
3,230 m	3,230 m	3,230 m	
1,659 sh	1,655 sh	1,648 S	υas O–C–O
1,620 S	1,625 S	1,618 S	υ C=C
1,600 S	1,596 S	1,603 S	υ C=N
1,557 m	1,559 s	1,559 s	δ N–H
1,517 m	1,517 s	1,515s	υ C–N
1,450 w	1,448 w	1,448 w	
1,358 w	1,345 w	1,344 m	υs O–C–O
1,319 m	1,318 m	1,315m	
1,216 w	1,211 m	1,211 m	δip C–H
1,012 w	1,012 m	1,012 m	υs C–O
800 w	790 w	797 m	δ O–C–O
830 w	819 w	819 m	
529 w	523 w	523 m	δop C–H
489 w	486 w	486 m	

[a] Relative intensities; S: very strong; s: strong; m: medium; w: weak; sh: shoulder.

Table S8. FTIR peaks (cm^{-1}) and corresponding vibration modes for aeroCo, xeroCo and single crystals of [Ni(ox)(4-apy)$_2$]$_n$ [a].

aeroNi	xeroNi	[Ni(ox)(4-apy)$_2$]$_n$	Vibration Mode
3,440 s	3,445 s	3,435 s	
3,347 s	3,355 s	3,355 s	υ N–H
3,227 m	3,220 m	3,225 m	
1,682 sh	1,682 sh	1,655 S	υas O–C–O
1,622 S	1,615 S	1,625 S	υ C=C
1,577 sh	1,580 sh	1,605 S	υ C=N
1,562 s	1,562 s	1,560 s	δ N–H
1,517 s	1,517 s	1,515s	υ C–N
1,450 w	1,450 w	1,445 w	
1,360 m	1,352 m	1,345 m	υs O–C–O
1,322 s	1,315 m	1,315m	
1,210 m	1,210 m	1,211 m	δip C–H
1,022 m	1,022 m	1,010 m	υs C–O
797 w	797 w	795 m	δ O–C–O
827 w	827 w	819 m	
527 w	529 w	530 m	δop C–H
490 w	482 w	487 m	

[a] Relative intensities; S: very strong; s: strong; m: medium; w: weak; sh: shoulder.

4. Pores Size Distribution (BJH)

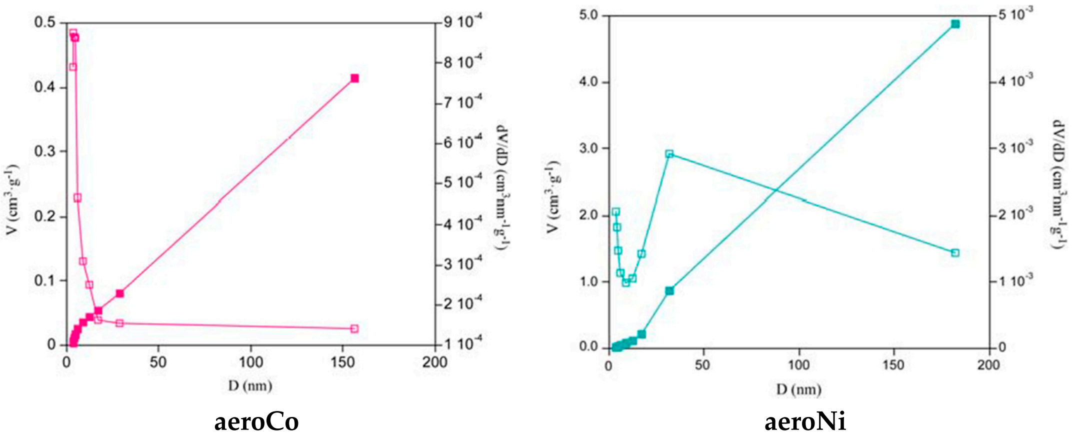

Figure S4. BJH pore size distributions for aeroCo and aeroNi (filled squares: cumulative pore volume; open squares: derivative pore volume).

© 2016 by the authors; licensee MDPI, Basel, Switzerland. This article is an open access article distributed under the terms and conditions of the Creative Commons by Attribution (CC-BY) license (http://creativecommons.org/licenses/by/4.0/).
